# Why Not

**Published:** 1914-07

**Authors:** 


### Why Not.

"We guard our business with a great deal of care. If a group of men desire to form a partnership, they must secure permission from the Legislature; if a man or a group of men desire to carry on a municipal trade, they must secure a license; if a man in the city of Chicago desires to carry on only an insignificant street trade, he must vouch for his responsibility at the city hall. If this same man desires to marry, he goes down to the city hall alone and gets a license. He may be a degenerate or an epileptic, or the diseases of the social evil may be coursing through his veins. These people are allowed to marry and propagate their kind, and to pass on to succeeding generations the physical, mental and moral deficiencies which they possess. We are giving much time and attention to the question of environment. We should give much attention to nurture. In face of the fact that we safeguard marriage by no laws and by no public attention, is it strange that we have in our public institutions three million abnormal people, costing this nation two hundred million a year for their care, and that they are increasing far and beyond the proportion of the increase of the population in this country, large as that increase is? The demand for a health certificate before marriage will come about by legislation, and this is effective just as it is backed up by public opinion. Public opinion today is not well informed with regard to the need of safeguarding marriage selection. Education of the public is the great hope, and the greatest agent for education in this country today is the press."

The *Madison County Doctor* commenting upon the above, written by Dean Summer, suggests:

"But why wait for the law? Why not take the law into your own hands and demand the health certificate from the man who comes to you seeking the hand of your daughter? Send for the man who is the proposed husband and ask him to place in your hands a certificate from a reputable doctor, showing that he is free from any communicable disease. Safeguard the life and health of your daughter in this direction just as much as you would shield her from any other contagion. You would not think of allowing her to marry a man that gave evidences of the more common contagious diseases, then why not protect her to the best of your ability from a condition that is immeasurably worse? Another thought comes in right here. Do you think you have done your whole duty to your child if you neglect to take this precaution? Dean Summer did not wait for any law. In the spring of 1912 he announced that beginning with Easter of that year no marriage ceremony would be solemnized in the Cathedral without the presentation of a health certificate by both of the contracting parties, which rule has been strictly enforced ever since. A month ago the dean reported that more marriage ceremonies had been celebrated at the Cathedral than ever before, showing that the demand for this certificate is not a bar to marriages contracted in good faith. Since this plan was inaugurated at Chicago, many clergymen at various places and of different denominations have adopted the same rule and have refused to officiate unless this certificate was produced. It can be done. Do not wait for the law. Act in a personal capacity."

